# Supplementary material for: Nanophysiology approach reveals diversity in calcium microdomains across zebrafish retinal bipolar ribbon synapses
Source: eLife. 2025 Dec 1;14:RP105875. doi: 10.7554/eLife.105875 (PMC12668674; doi:10.7554/eLife.105875)
Supplement: Figure 8—source data 1. — Table explaining how the data is presented in Figure 8A–C. [file elife-105875-fig8-data1.docx]

| **Cell** | **Ribbon** | **Measurement Number** | **Data Processing** | **Data Presentation** |
| --- | --- | --- | --- | --- |
| Cell 1 | Ribbon 1 | Measurement 1 | All measurements from ribbon 1 are averaged. | Cell A |
|  |  | Measurement 2 |  |  |
|  |  | Measurement 3 |  |  |
|  | Ribbon 2 | Measurement 1 | All measurements from ribbon 2 are averaged. |  |
|  |  | Measurement 2 |  |  |
|  |  | Measurement 3 |  |  |
|  | Ribbon 3 | Measurement 1 | All measurements from ribbon 3 are averaged. |  |
|  |  | Measurement 2 |  |  |
|  |  | Measurement 3 |  |  |
| Cell 2 | Ribbon 1 | Measurement 1 | All measurements from ribbon 1 are averaged. | Cell B |
|  |  | Measurement 2 |  |  |
|  |  | Measurement 3 |  |  |
|  | Ribbon 2 | Measurement 1 | All measurements from ribbon 2 are averaged. |  |
|  |  | Measurement 2 |  |  |
|  |  | Measurement 3 |  |  |
|  | Ribbon 3 | Measurement 1 | All measurements from ribbon 3 are averaged. |  |
|  |  | Measurement 2 |  |  |
|  |  | Measurement 3 |  |  |

**Figure 8–source data 1. Data presentation for ribbon variability between cells.**

Table explaining how the data is presented in **Figure 8A-C**.
