# Supplementary material for: Nanophysiology approach reveals diversity in calcium microdomains across zebrafish retinal bipolar ribbon synapses
Source: eLife. 2025 Dec 1;14:RP105875. doi: 10.7554/eLife.105875 (PMC12668674; doi:10.7554/eLife.105875)
Supplement: Figure 8—source data 2. — Table explaining how the data is presented in Figure 8D–F. [file elife-105875-fig8-data2.docx]

| **Cell** | **Ribbon** | **Measurement Number** | **Data Presentation** |
| --- | --- | --- | --- |
| Cell 1 | Ribbon 1 | Measurement 1 | Cell A |
|  |  | Measurement 2 | Cell A |
|  |  | Measurement 3 | Cell A |
|  | Ribbon 2 | Measurement 1 | Cell B |
|  |  | Measurement 2 | Cell B |
|  |  | Measurement 3 | Cell B |
|  | Ribbon 3 | Measurement 1 | Cell C |
|  |  | Measurement 2 | Cell C |
|  |  | Measurement 3 | Cell C |

**Figure 8–source data 2. Data presentation for ribbon variability within individual cells.**

Table explaining how the data is presented in **Figure 8D-F**.
