# Supplementary material for: Nanophysiology approach reveals diversity in calcium microdomains across zebrafish retinal bipolar ribbon synapses
Source: eLife. 2025 Dec 1;14:RP105875. doi: 10.7554/eLife.105875 (PMC12668674; doi:10.7554/eLife.105875)
Supplement: Supplementary file 1. — There were significant differences between proximal vs distal measured as ΔF/Frest, in all conditions, as found through paired-sample t-test analysis performed on RStudio. Differences were smaller between proximal vs distal Ca2+ signals in 0.2 mM, 2 mM, and 10 mM EGTA conditions, but more prominent with 2 mM BAPTA (0.2 mM EGTA: proximal vs distal: p=0.0027, 2 mM EGTA: proximal vs distal: p=0.034, 10 mM EGTA: proximal vs distal p=0.00013, 2 mM BAPTA: proximal vs. distal: p=0.0073, n=22). [file elife-105875-supp1.docx]

|  | Proximal (Δ*F*/*F*_rest_) | Distal (Δ*F*/*F*_rest_) |
| --- | --- | --- |
| 0.2 mM EGTA | 5.5 ± 0.9  (N=30) | 3.3 ± 0.8  (N=30) |
| 2 mM EGTA | 6.6± 0.8  (N=21) | 4 ± 0.8  (N=21) |
| 10 mM EGTA | 3.5 ± 0.4  (N=43) | 1.8 ± 0.2  (N=43) |
| 2 mM BAPTA | 3.6 ± 1  (N=20) | 0.8 ± 0.2  (N=20) |

**Supplementary File 1**. **Effect of exogenous Ca^2+^ chelators alter Ca^2+^ signals gradient along synaptic ribbon measured with Cal520LA-RBP.** There were significant differences between proximal vs distal measured as Δ*F*/*F*_rest_, in all conditions as found through paired-sample t-test analysis performed on RStudio. Differences were smaller between proximal vs distal Ca2+ signals in 0.2 mM, 2 mM, and 10 mM EGTA conditions, but more prominent with 2 mM BAPTA (0.2 mM EGTA: proximal vs distal: p = 0.0027, 2 mM EGTA: proximal vs distal: p = 0.034, 10 mM EGTA: proximal vs distal p = 0.00013, 2 mM BAPTA: proximal vs. distal: p = 0.0073, n = 22).
