# Supplementary material for: Nanophysiology approach reveals diversity in calcium microdomains across zebrafish retinal bipolar ribbon synapses
Source: eLife. 2025 Dec 1;14:RP105875. doi: 10.7554/eLife.105875 (PMC12668674; doi:10.7554/eLife.105875)
Supplement: Supplementary file 2. — There were significant differences between proximal vs distal measured as ΔF/Frest, in all conditions as found through paired-sample t-test analysis performed on RStudio. Differences were smaller between proximal vs distal Ca2+ signals in 0.2 mM EGTA and 2 mM EGTA conditions, but more prominent with 10 mM EGTA, and further enhanced with 2 mM BAPTA (0.2 mM EGTA: proximal vs distal p=0.00135, n=19; 2 mM EGTA: proximal vs distal p=7.4∙10–4, n=23; 10 mM EGTA: proximal vs distal p=1.4∙10–5, n=29; 2 mM BAPTA: proximal vs distal p=0.0046, n=22). [file elife-105875-supp2.docx]

|  | Proximal (Δ*F*/*F*_rest_) | Distal (Δ*F*/*F*_rest_) |
| --- | --- | --- |
| 0.2 mM EGTA | 3.0 ± 0.4  (N=19) | 1.9 ± 0.3  (N=19) |
| 2 mM EGTA | 3.9 ± 0.4  (N=23) | 2.8 ± 0.2  (N=23) |
| 10 mM EGTA | 4.4 ± 0.4  (N=29) | 2.2 ± 0.2  (N=29) |
| 2 mM BAPTA | 1.5 ± 0.3  (N=22) | 0.5 ± 0.1  (N=22) |

**Supplementary File 2**. **Effect of exogenous Ca^2+^ chelators alters the Ca^2+^ signal gradient along the synaptic ribbon measured with Cal520H-RBP.** There were significant differences between proximal vs distal measured as Δ*F*/*F*_rest_, in all conditions as found through paired-sample t-test analysis performed on RStudio. Differences were smaller between proximal vs distal Ca^2+^ signals in 0.2 mM EGTA and 2 mM EGTA conditions, but more prominent with 10 mM EGTA, and further enhanced with 2 mM BAPTA (0.2 mM EGTA: proximal vs distal p = 0.00135, n = 19; 2 mM EGTA: proximal vs distal p = 7.4∙10^-4^, n = 23; 10 mM EGTA: proximal vs distal p = 1.4∙10^-5^, n = 29; 2 mM BAPTA: proximal vs distal p = 0.0046, n = 22).
