# Supplementary material for: Nanophysiology approach reveals diversity in calcium microdomains across zebrafish retinal bipolar ribbon synapses
Source: eLife. 2025 Dec 1;14:RP105875. doi: 10.7554/eLife.105875 (PMC12668674; doi:10.7554/eLife.105875)
Supplement: Supplementary file 3. — Simulations were performed assuming an endogenous buffer with a total concentration of either 1.4 mM total resting buffering capacity of 720 Oesch and Diamond, 2011; Burrone et al., 2002; Coggins and Zenisek, 2009, or a lower concentration of 200 µM corresponding to a buffering capacity of 100. Simulations in Figure 7 assumes immobile endogenous buffer, while this file assumes a typical value of buffer mobility of 0.05 µm2/ms. The Ca2+ clearance parameters are adapted from Graydon et al., 2011; Jarsky et al., 2010; Mennerick and Matthews, 1996; Singer and Diamond, 2003; Snellman et al., 2009; Von Gersdorff and Mathews, 1994; Augustine et al., 1991. Note that flux units of (µM µm)/ms are equivalent to 10–21 mol/(µm2ms)=602 ions/(µm2ms). Properties of EGTA and BAPTA (not listed here) are summarized in Burrone et al., 2002. [file elife-105875-supp3.docx]

| Symbol | Value / units | Description |
| --- | --- | --- |
| *C* | µM | Ca^2+^ concentration, [Ca^2+^] |
| *B* | µM | Free (unbound) buffer concentration, [B] |
| *B^*^* | µM | Bound buffer concentration, [CaB] |
| *C*_0_ | 0.1 µM | Resting background [Ca^2+^] |
| *D_C_* | 0.22 µm^2^/ms | Intracellular diffusivity of Ca^2+^ ions |
| *D_B_* | 0 or 0.05 µm^2^/ms | Buffer diffusivity (two values used) |
| *B_total_* | 200 µM or 1.4 mM | Total buffer concentration (two values used) |
| *k^+^* | 0.1 (µM ms)^-1^ | Buffer-Ca^2+^ binding rate |
| *k^-^* | 0.2 ms^-1^ | Buffer-Ca^2+^ unbinding rate |
| *K_D_* | 2 µM | Buffer affinity |
| *A*_NCX_ | 0.4 µM µm/ms | Maximal extrusion rate by NCX exchanger |
| *A*_P_ | 0.03 µM µm/ms | Maximal extrusion rate by SERCA & PMCA pumps |
| *K*_NCX_ | 1.5 µM | Affinity of NCX exchanger |
| *K*_P_ | 0.3 µM | Affinity of SERCA/PMCA pumps |

**Supplementary File 3**: Model parameters for Ca^2+^ diffusion, buffering, and clearance. Simulations were performed assuming an endogenous buffer with a total concentration of either 1.4 mM (total resting buffering capacity of 720 ^6-8^, or a lower concentration of 200 µM corresponding to a buffering capacity of 100. Simulations in **Fig. 7** assumes immobile endogenous buffer, while **Supplementary** **Fig. 4** assumes a typical value of buffer mobility of 0.05 µm^2^/ms. The Ca^2+^ clearance parameters are adapted from ^9-15^. Note that flux units of (µM µm)/ms are equivalent to 10^-21^ mol/(µm^2^ms) = 602 ions/(µm^2^ms). Properties of EGTA and BAPTA (not listed here) are summarized in ^7^.
